# Comparative efficacy and safety of POLARx versus Arctic front advance pro cryoballoon systems for pulmonary vein isolation in atrial fibrillation: an updated systematic review and meta-analysis

**DOI:** 10.3389/fcvm.2025.1625399

**Published:** 2025-08-18

**Authors:** Muhammad Fawad Tahir, Muhammad Hamza Shuja, Ramish Hannat, Fabeeha Shaheen, Amna Sayeed, Ayesha Shaukat, Faiza Rajput, Muhammad Ahmed Zahoor, Kinza Hamid, Areeba Khan, Aymen Azan Ahmed, Syeda Asfiha Wasiq, Huda Naeem, Bakhtawar Shaikh, Muhammad Omar Larik, Raheel Ahmed, Mustafa Shehzad, Mah I. Kan Changez, Muhammad Usman Haider, Tanesh Ayyalu

**Affiliations:** ^1^ Department of Medicine, H.B.S Medical and Dental College, Islamabad, Pakistan; ^2^ Department of Cardiology, Rawalpindi Institute of Cardiology, Rawalpindi, Pakistan; ^3^ Department of Medicine, Dow University of Health Sciences, Karachi, Pakistan; ^4^ Department of Medicine, Services Institute of Medical Sciences (SIMS), Lahore, Pakistan; ^5^ Department of Medicine, Jinnah Sindh Medical University, Karachi, Pakistan; ^6^ Department of Medicine, Karachi Medical and Dental College, Karachi Metropolitan University, Karachi, Pakistan; ^7^ Department of Medicine, Akhtar Saeed Medical and Dental College, Lahore, Pakistan; ^8^ Department of Medicine, Peshawar Medical College, Peshawar, Pakistan; ^9^ Department of Cardiology, Dow International Medical College, Karachi, Pakistan; ^10^ National Heart and Lung Institute, Imperial College London, London, United Kingdom; ^11^ Department of Medicine, Hackensack University Medical Center, Hackensack, NJ, United States; ^12^ Department of Cardiac Surgery, Yale University, New Haven, CT, United States; ^13^ Department of Internal Medicine, Geisinger Health System, Pennsylvania, PA, United States; ^14^ Department of Cardiology, MedStar Heart and Vascular Institute, Washington, DC, United States

**Keywords:** cryoballoon ablation, atrial fibrillation, pulmonary vein isolation, POLARx, Arctic front advance

## Abstract

**Background:**

Cryoballoon (CB) ablation is a well-established treatment for atrial fibrillation (AF). The Arctic Front Advance Pro™ (AFA-Pro) system, in use for over a decade, has demonstrated consistent efficacy and safety. Recently, the POLARx™, a novel CB system, has gained attention due to its advanced design and comparable clinical outcomes.

**Methods:**

We conducted a systematic review and meta-analysis of studies comparing AFA-Pro and POLARx in patients undergoing CB ablation for AF. Outcomes included procedural parameters (procedure time, ablation time, fluoroscopy time, balloon nadir temperature) and safety endpoints such as phrenic nerve palsy (PNP) and Stroke rates.

**Results:**

There were no significant differences between POLARx and AFA in acute PVI success (OR = 0.49, P = 0.24), procedure time (MD = 4.40, P = 0.14), ablation time (SMD = 0.12, P = 0.10), fluoroscopy time (MD = 0.34, P = 0.67), freezing time and stroke rates. POLARx showed a significant advantage in balloon nadir temperature (P < 0.00001) and demonstrated longer time to isolation (TTI) in the RSPV (MD = 5.49, P = 0.05) and RIPV (MD = 4.54, P = 0.04), while TTI was similar for the LSPV and LIPV. However, POLARx was associated with a higher risk of PNP (OR = 1.87, P = 0.007).

**Conclusion:**

POLARx demonstrates lower balloon nadir temperature but is associated with a higher risk of phrenic nerve palsy compared to AFA-Pro. These findings provide important insights into the procedural efficiency and safety profiles of these cryoablation systems.

**Systematic Review Registration:**

identifier CRD42024588371.

## 1 Introduction

Atrial Fibrillation is a prevalent cardiac arrhythmia marked by rapid and irregular electrical activity in the atria, leading to an increased risk of thromboembolic events (1). According to the 2023 ACC/AHA/ACCP/HRS guideline for diagnosing and managing atrial fibrillation, catheter ablation of AF is classified as a Class 1 indication for first-line therapy in selected patients. Recent studies have shown that catheter ablation is more effective than drug therapy for rhythm control. Cryoballoon (CB) ablation has become increasingly popular due to its ease of use, safety and effectiveness in achieving pulmonary vein isolation (2). The Arctic Front Advance system by Medtronic (USA) is a widely recognized technology, with the Arctic Front Advance Pro being its latest fourth-generation version (3). However, the field of cryoballoon ablation is advancing with the introduction of the POLARx system by Boston Scientific (USA) (4). Preliminary studies suggest that POLARx may offer lower minimal temperatures and shorter procedure time compared to earlier Arctic Front systems (5).

Several centers have reported their clinical experiences comparing the two balloon systems, focusing on acute procedural efficacy, balloon nadir temperature, incidence of phrenic nerve palsy, and acute recurrence of atrial fibrillation (4, 6). A previous meta-analysis by Assaf et al. (7) indicated that the acute outcomes of the POLARx system are comparable to those of the AFA-Pro, despite POLARx demonstrating lower balloon nadir temperatures. Notably, the POLARx system showed a higher rate of time to isolation recordings in the inferior pulmonary veins.

Recently, additional studies have emerged that compare both balloon systems since the publication of that meta-analysis (8, 9). Therefore, we aim to conduct an updated meta-analysis to compare the efficacy, safety, and procedural outcomes of the AFA-Pro and POLARx cryoballoon systems. Our evaluation will focus on procedural efficacy, ablation time, fluoroscopy time, balloon nadir temperature, incidence of phrenic nerve palsy, acute pulmonary vein isolation success, and stroke. Furthermore, we will assess novel outcome measures, including freezing characteristics and TTI recordings in all four pulmonary veins (LIPV, LSPV, RIPV, and RSPV). As the POLARx cryoablation system continues to gain widespread adoption, this updated meta-analysis aims to provide critical insights into its comparative performance, efficacy and safety with the AFA-Pro system.

## 2 Methodology

This systematic review and meta-analysis were conducted in accordance with the “Preferred Reporting Items for Systematic Reviews and Meta-Analyses” (PRISMA) guidelines and followed the methodology framework established by the Cochrane Collaboration. The protocol for this review was prospectively registered with PROSPERO (ID: CRD42024588371).

### 2.1 Data sources and search strategy

A comprehensive search was conducted across several electronic databases, including PubMed, Google Scholar, ScienceDirect, and Clinicaltrials.gov from December 1, 2019 till August 31, 2024. The search strategy aimed to identify studies comparing the AFA-Pro and POLARx cryoballoon systems in patients undergoing catheter ablation for paroxysmal or persistent atrial fibrillation. Keywords used in the search included “AFA-Pro,” “POLARx,” “cryoballoon,” “atrial fibrillation,” “pulmonary vein isolation,” and related terms. No filters were applied based on language, year of publication, or study design. A full search strategy is provided in Supplementary Table 1.

### 2.2 Study selection and eligibility criteria

Duplicates were removed using EndNote Reference Manager, and two independent reviewers (M.H.S. and F.T.) screened titles and abstracts against predefined eligibility criteria. Full-text articles that met the inclusion criteria were reviewed in detail, with disagreements resolved by a third reviewer (F.S.). In accordance with the Cochrane Collaboration methodology, the study question was structured using the PICO format:

• Population: adults aged 18 years or older with paroxysmal or persistent atrial fibrillation (AF) undergoing pulmonary vein isolation.
• Intervention: cryoablation using the POLARx cryoballoon system.
• Comparator: cryoablation using the AFA-PRO cryoballoon system; and
• Outcomes: procedural efficacy (defined as successful pulmonary vein isolation during the index procedure), acute complications (including phrenic nerve palsy and stroke), and intra-procedural metrics (such as ablation time, fluoroscopy time, and balloon nadir temperature).

Eligible studies had to report procedural efficacy as the acute outcome of the cryoballoon ablation procedure, defined by the achievement of pulmonary vein isolation during the index procedure, along with acute complications, including the incidence of phrenic nerve palsy and stroke occurrence. Most of the included studies in our meta-analysis included stroke as a periprocedural complication. Other key intra-procedural parameters, including ablation and fluoroscopy times, as well as balloon nadir temperature, were also reported. Key exclusion criteria included prior left atrial ablation or surgery, reversible causes of atrial fibrillation, severe mitral regurgitation, reduced left ventricular ejection fraction (<35%), or NYHA class III/IV heart failure. Studies were excluded if they did not compare cryoballoon systems or were not published in English.

### 2.3 Data extraction and quality assessment

Baseline patient characteristics extracted from each included study comprised age, sex, type of atrial fibrillation (AF), presence of hypertension, diabetes, coronary artery disease, and left atrial size. Outcome data collected at the patient level included acute pulmonary vein isolation (PVI) success, operation time, fluoroscopy duration, ablation time, occurrence of phrenic nerve palsy (PNP), and incidence of stroke or transient ischemic attack (TIA). When reported, the following data was obtained for each pulmonary vein (PV): minimum esophageal temperature, balloon nadir temperature, isolation achieved with the first freeze, time-to-isolation (TTI) recording, and TTI values.

The quality of the included studies in this meta-analysis was assessed using two established tools to evaluate the risk of bias: the Revised Cochrane Risk-of-Bias Tool for Randomized Trials (ROB-2) and the Newcastle-Ottawa Scale (NOS). These tools were applied systematically to assess the methodological quality of both randomized controlled trials (RCTs) and non-randomized studies. Two independent reviewers (A.K. and M.H.S.) conducted the risk-of-bias evaluations, with any discrepancies resolved through discussion. The ROB-2 tool is explicitly used for RCTs, assessing five key domains: bias from the randomization process (e.g., sequence generation and allocation concealment), performance bias (deviations from the intended interventions), attrition bias (missing data), detection bias (measurement of outcomes), and reporting bias (selective outcome reporting), with each domain rated as low, high, or unclear risk (10). In contrast, the NOS was used to evaluate cohort and case-control studies in non-randomized designs, focusing on three areas: selection of study groups (e.g., representativeness and exposure ascertainment), comparability of groups (e.g., controlling for confounders), and outcome assessment (e.g., adequacy of follow-up), with studies awarded points based on these criteria; a score of 7 or higher typically suggests a low risk of bias (11).

### 2.4 Statistical analysis

For statistical analysis, we used RevMan (Version 5.4.1) to pool data from the selected studies. Using the meta-accelerator tool (12), medians and interquartile ranges were used to estimate means and standard deviations (13). Meta-analyses were performed using the random-effects model according to the DerSimonian and Laird method to account for potential heterogeneity across studies (14). Continuous outcomes were expressed as mean differences (MD) or standardized mean differences (SMD), depending on measurement units. For dichotomous outcomes, we calculated odds ratios (ORs) with 95% confidence intervals (CIs). A *p*-value of <0.05 was considered statistically significant. The *I*^2^ statistical test was used to assess heterogeneity between studies. Values of 0%–25% were considered low heterogeneity, 25%–50% moderate, 50%–75% substantial, and >75% high heterogeneity. In cases of significant heterogeneity, we performed sensitivity analyses to test the robustness of the results (15). Sensitivity analysis was performed using a leave-one-out approach, whereby each study was sequentially excluded to evaluate its impact on the overall results. Publication bias was evaluated through both visual and statistical approaches. We generated funnel plots for each pooled analysis and inspected them for asymmetry suggestive of small-study effects. In addition, we applied Begg's rank-correlation test and Egger's weighted regression test to quantify funnel-plot asymmetry; a two-tailed *p*-value <0.05 on either test was considered indicative of potential publication bias. All publication-bias analyses were conducted in R (meta and metafor packages, version 4.3.3).

## 3 Results

**Figure 1:**
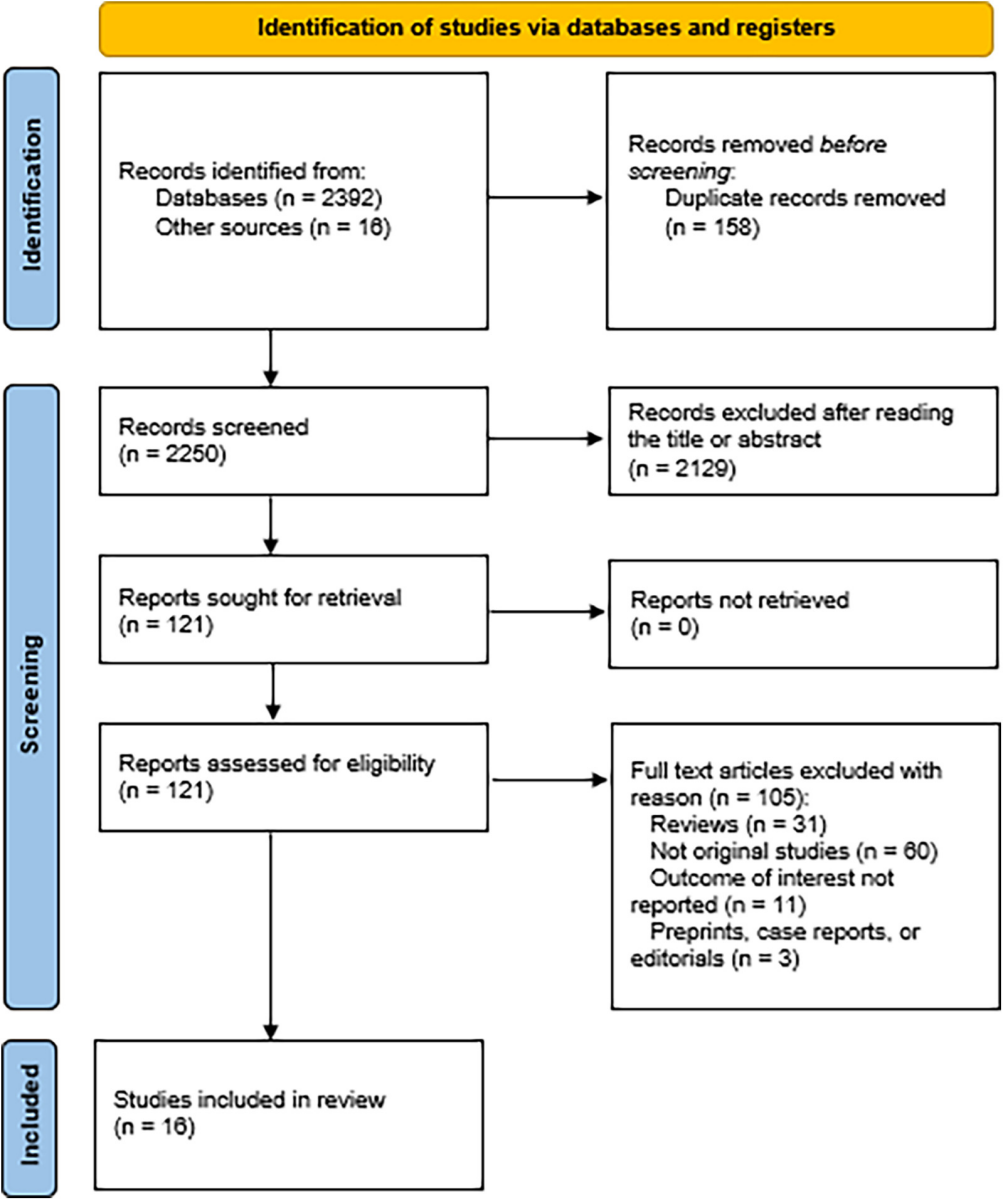
PRISMA flowchart illustrating the screening process.

A total of 2,408 studies were identified after a step-by-step screening process. Following duplicate study removal, assessment of abstracts based on inclusion and exclusion criteria, and a thorough review of the full texts, 16 studies were selected to be included in this analysis (4–6, 8, 9, 16–26). The PRISMA flowchart illustrates the details in Figure 1.

### 3.1 Study characteristics

A total of 6,583 individuals participated in the 1 RCT, and 15 cohorts were included in this meta-analysis (4–6, 8, 9, 16–26). Of these, 2,943 belonged to the POLARx group, and 3,640 were in the AFA group. The year of publication ranged from December 1, 2019 to August 1, 2024. The baseline characteristics across studies reveal a predominantly middle-aged to elderly population with a male predominance in both POLARx and AFA Pro groups. Sample sizes ranged widely, from 50 to 2,555 participants, ensuring diverse study designs. The prevalence of paroxysmal atrial fibrillation (PAF) varied between groups, but gender and age were generally well-balanced within individual studies. Baseline and study characteristics are further detailed in Tables 1, 2.

**Table 1:** Baseline clinical and study characteristics of the included study population.

Study name	Sample size (*n*)		AGE (Mean ± SD)		Percentage of males		Percentage of PAF		Percentage of HTN		Percentage of CAD		Left atrial volume index (Mean ± SD) Or Median (Q1–Q3)		BMI/Obesity		Structural Heart Disease		CHA2DS2VASc		Anticoagulation Regimen (pre-)		Heart Failure (HF)		Left Ventricular Ejection Fraction (LVEF)	
	Total	Polarx/AFA pro	POLARx	AFA Pro	POLARx	AFA Pro	POLAR x	AFA Pro	POLAR x	AFA Pro	POLAR x	AFA Pro	POLAR x	AFA Pro	POLAR x	AFA Pro	POLAR x	AFA Pro	POLAR x	AFA Pro	POLAR x	AFA Pro	POLAR x	AFA Pro	POLAR x	AFA Pro
Creta et al. (6)	80	40/40	62.8 ± 11.7	65.0 ± 12.1	57.5	55	68	50	42.5	35	N/A	N/A	38.3 ± 4.1 Mm	40.2 ± 6.0 mm	N/A	N/A	—	—	N/A	N/A	On uninterrupted (VKA) or (DOAC)	On uninterrupted (VKA) or (DOAC)	N/A	N/A	80%	77.5%
Knecht et al. (2021)	80	40/40	65 ± 11	66 ± 9	65	65	58	70	50	50	28	18	36 ± 12 ml/m^2^	41 ± 13 ml/m^2^	28 ± 7 kg/m2	27 ± 4 kg/m2	—	—	N/A	N/A	OAC post-procedure	OAC post-procedure	13%	3%	57 ± 12%	58 ± 7%
Kochi et al. (4)	70	20/50	63 ± 59.6	61 (53–70.2)^a^	60	84	95	94	60	30	5	18	36 (30–41) ml/m^2^	33 (31–43) ml/m^2^	N/A	N/A	—	—	1.5 (0–2.0)^a^	1.0 (0–3.0)^a^	—	—	N/A	N/A	60%	62%
Mojica et al. (16)	60	30/30	57.47 ± 15.24	53.53 ± 16.24	66	60	100	100	33	30	3	10	31.5 ± 8.23 ml/m^2^	31.87 ± 7.31 ml/m^2^	N/A	N/A	—	—	1.27 ± 1.33	1.13 ± 1.40	OAC	OAC	10%	3%	N/A	N/A
Moser et al. (17)	100	50/50	64.5 (57,74)^a^	67 (55,75)^a^	82	62	56	40	60	74	22	28	N/A	N/A	27 (24,32)^a^	28 (24,31)^a^	—	—	2 (1.25,3)^a^	3 (2,4)^a^	oral VKA) or NOACs interrupted 12 h prior procedureru	oral VKA () or NOACs interrupted 12 h prior to procedure	34%	28%	57%	55%
Tilz et al. (5)	50	25/25	68 (62,73)^a^	69 (59,75)^a^	52	68	48	36	80	72	16	24	25 (25,30)^a^ ml/m2	29 (25,35)^a^ ml/m2	N/A	N/A	NA	NA			LMWH was administered in patients with and INR <2.0. New Oral anticoagulants were re-initiated 6 h post ablation.	LMWH was administered in patients with and INR <2.0. New Oral anticoagulants were re-initiated 6 h post ablation.	Congestive heart failure: 2 (8%)	Congestive heart failure: 4 (16%)	N/A	N/A
Yap et al. (25)	110	57/53	61 (57, 66)	64 (57, 70)	57.9	67.9	75.4	75.5	31.6	58.5	14.0	9.4	41 (36, 44)^a^ LEFT ATRIAL DIAMETER	41 (37,43)^a^ LEFT ATRIAL DIAMETER	N/A	N/A	CABG: 2 (3.5%)	CABG: 1 (1.9%)	1 (1,2)^a^	2 (0,3)^a^	DOAC: 57 (100.0%). All patients received oral anticoagulation for at least 4 weeks prior to ablation	DOAC: 48 (90.6%). Vitamin K Antagonist: 3 (5.7%). All patients received oral anticoagulation for at least 4 weeks prior to ablation	N/A	N/A	63 (60–65)^a^%	60 (60–65)^a^%
Bisignani et al. (22)	80	40/40	66.6 ± 12.6	62.8 ± 11.9	55	65	0	0	77.5	70	N/A	N/A	48.1 ± 9.7	49 ± 8.3	N/A	N/A	N/A	N/A	2.6 ± 1.4	2.2 ± 1.5	N/A	N/A	N/A	N/A	52.5 ± 6.7%	55 ± 7.5%
Guckel et al. (23)	687	86/601	61.3 ± 11.1	59.2 ± 20.8	69	72	58	58	57	55	N/A	N/A	39.1 ± 6.7 ml/m^2^	39.2 ± 7.3 ml/m^2^	29.6 ± 8.0 kg/m^2^	27.9 ± 4.6 kg/m^2^	13%	12%	—	—	Phenprocoumon was continued aiming an INR between 2.0 and 3.0. DOAC stopped one hour before ablation	Phenprocoumon was continued aiming an INR between 2.0 and 3.0. DOAC stopped one hour before ablation	N/A	N/A	52.9 ± 3.7%	53.5 ± 4.8%
Heeger et al. (21)	205	103/102	68.7 ± 10.2	65.7 ± 12	54	62	51	41	74	70	28	26	32.9 ± 11.4 ml/m^2^	31.7 ± 9.8 ml/m^2^	N/A	N/A	N/A	N/A	—	—	Procedure conducted under therapeutic INR values between 2 and 3 for VKAs, morning dose omitted for NOACs	Procedure conducted under therapeutic INR values between 2 and 3 for VKAs, morning dose omitted for NOACs	11%	15%	N/A	N/A
Honarbakhsh et al. (24)	1,688	844/844	61.4 ± 12.8	62.3 ± 11.1	65.0	67.5	65.5	71.7	30.9	33.9	7.3	8.1	42.6 ± 4.7 mm	42.1 ± 6.7 mm	N/A	N/A	10	10.9	N/A	N/A	Warfarin and DOAC	Warfarin and DOAC			LVEF > 55% 74.8%	LVEF > 55% 77.0%
Menger et al. (18)	122	61/61	63.3 ± 11.8	64.8 ± 12.0	62.3	55.7	63.9	73.8	N/A	N/A	N/A	N/A	3.9 ± 0.7 cm	3.8 ± 0.6 cm	29.5 ± 5.0 kg/m^2^	29.2 ± 6.3 kg/m^2^	—	—	2.3 ± 1.6	2.5 ± 1.6	52 (85.3%)	58 (95.1%)	N/A	N/A	53.3 ± 7.8%	53.4 ± 5.0%
Tanese et al. (20)	267	137/130	63.3 ± 10.7	63.2 ± 11.1	59	62	100	100	42	49	7	11	N/A	N/A	N/A	N/A	DCM1% HCM2% VALVULAR 0%	DCM2% HCM1% VALVULAR 1%	—	—	Uninterrupted anticoagulation	Uninterrupted anticoagulation	3%	3%	N/A	N/A
Knappe et al. (9)	228	114/114	67.2 ± 10.1	68.0 ± 11.1	62.3	62.3	62.3	48.2	80.7	77.2	25.4	24.6	N/A	N/A	27.2 ± 3.8 kg/m2	27.7 ± 4.5 kg/m2	—	—	2.7 ± 1.7	2.9 ± 1.5	For patients receiving new oral anticoagulants the morning dose of the procedure was withheld.for patients takingvit k antagonist, procedure was conducted only when inr was between2 and 3	For patients receiving new oral anticoagulants the morning dose of the procedure was withheld.for patients takingvit k antagonist, procedure was conducted only when inr was between2 and 3	9.6%	10.5%	58.1 (55.7,63.5)^a^%	57.8 (55.1,63.6)^a^%
Reichlin et al. (8)	201	99/102	62.2 ± 10.1	62.2 ± 9.3	65	76	100	100	48	55	N/A	N/A	34 ± 12 ml/m^2^	34 ± 10 ml/m^2^	26.9 ± 4.8 kg/m^2^	26.9 ± 4.8 kg/m^2^	—	—	1.8 ± 1.6	1.6 ± 1.4	Continuous vit k antagonist or DOAC in 78%patients for > _4weeks	Continuous vit k antagonist or DOAC in 84%patients for > _4weeks	N/A	N/A	60 ± 5%	61 ± 6%
Tachibana et al. (19)	2,555	1,197/1,358	68.2 ± 10.8	69.4 ± 10.9	67.7	68.5	64.6	66.1	N/A	N/A	N/A	N/A	38.6 ± 6.9 mm	39.1 ± 6.3 mm	23.9 ± 3.9 kg/m^2^	24.3 ± 4.0 kg/m^2^	154 (12.9%)	168 (12.4%)	2.2 ± 1.5	2.3 ± 1.5	According to HRS guidelines	According to HRS guidelines	—	—	60.9 ± 10.7%	62.6 ± 10.9%

^a^ Data is given in median (Q1–Q3).

PAF, paroxysmal atrial firillation; HTN, hypertension; CAD, coronary artery disease.

**Table 2:** Study characteristics of the included study population.

Study (year)	Country	Study type	Design	Freezing protocol	Bonus freeze	Number of patients in POLARx Group	Number of patients in AFA-Pro group
Creta et al. (6)	UK	Prospective	Single center	Cryoballon Size: 28 mm Duration of Each Freeze: Standard: 180 s TTI-Guided Strategy: Operator discretion based on TTI, temperature lowering speed and nadir Bonus Freeze or Not: Not	No	40	40
Knecht et al. (2021)	Switzerland	Prospective	Multi-center	Cryoballon Size: 28 mm Duration of Each Freeze: Standard: 180–240 s TTI-Guided Strategy: TTI-guided Target TTI <60 s Bonus Freeze or Not: No	No	40	40
Kochi et al. (4)	Italy	Prospective	Single-center	Cryoballon Size: 28 mm Duration of Each Freeze: 180 to 300 s Bonus Freeze or Not: No	No	20	50
Mojica et al. (16)	Belgium	Retrospective	Single-center	Cryoballon Size: 28 mm Duration of Each Freeze: Single 180 s TTI-Guided Strategy: TTI-guided (or temperature <−40 °C within 1 min) Bonus Freeze or Not: Bonus freeze delivered if TTI or temperature <−40 °C not met within 1 min	Yes	30	30
Moser et al. (17)	Germany	Prospective	Single-center	Cryoballon Size: 28 mm Duration of Each Freeze: Standard: 180 s. If TTI is achieved, continued for 120 s TTI-Guided Strategy: TTI-based ablation protocol Bonus Freeze or Not: No	No	50	50
Tilz et al. (5)	Germany	Prospective	Single-center	Cryoballon Size: 28 mm Duration of Each Freeze: Standard: 180 s. If TTI ≥60 s, 180 s plus 180 s bonus TTI-Guided Strategy: TTI-based ablation protocol Bonus Freeze or Not: 180 s bonus freeze if TTI ≥60 s	Yes	25	25
Yap et al. (25)	Croatia, Germany, Netherlands	Prospective	Multi-center	Cryoballon Size: 28 mm Duration of Each Freeze: 180 s if TTI <60 s, otherwise 240 s TTI-Guided Strategy: TTI-guided Bonus Freeze or Not: No bonus freeze	No	57	53
Bisignani et al. (22)	Belgium	Prospective	Single centred	Cryoballon Size: 28 mm Duration of Each Freeze: Standard: 180 s. For LAPWI: 120 s. Target Temperature: ≤−40 °C within 60 s TTI-Guided Strategy: Based on temperature attainment (if not ≤−40 °C in 60 s) Bonus Freeze or Not: Extra freeze delivered if the target temperature not attained	NO	40	40
Denise Guckel et al. (2022)	germany	Retrospective	Single centred	Cryoballon Size: 28 mm Duration of Each Freeze: 2 × 180 s per vein Bonus Freeze or Not: No bonus freeze	No	86	601
Heeger et al. (21)	germany	Prospective	Single centred	Cryoballon Size: 28 mm Duration of Each Freeze: 180 s if TTI <60 s. 180 s plus 180 s bonus if TTI ≥60 s TTI-Guided Strategy: TTI-based approach Bonus Freeze or Not: Bonus freeze applied if TTI ≥60 s	Yes	103	102
Honarbakhsh et al. (24)	UK	Prospective	Multi-center	Cryoballon Size: 28 mm Duration of Each Freeze: Standard: 180 s. Extended to 240 s at the operator's discretion TTI-Guided Strategy: — Bonus Freeze or Not: Additional consolidating cryoablation at the operator's discretion	Yes	844	844
Menger et al. (18)	Germany		Single centred	Cryoballon Size: 28 mm Duration of Each Freeze: Standard: 180 s. Up to 240 s for late TTI or unsatisfactory temps TTI-Guided Strategy: TTI assessed rigorously Bonus Freeze or Not: No	No	61	61
Tanese et al. (20)	Belgium, France	Prospective	multi-centred	Cryoballon Size: 28 mm Duration of Each Freeze: Total freezing time set to 180–240 s. Freezing cycle aborted if TTI >60 s TTI-Guided Strategy: TTI-guided. Freeze aborted if TTI >60 s Bonus Freeze or Not: Bonus freeze systematically delivered if no stable PV potential assessed during cryo-application	Yes	137	130
Knappe et al. (9)	Germany	Retrospective	Single center	Duration of Each Freeze:240 s TTI-Guided Strategy: yes Bonus Freeze or Not:No	No	114	114
Reichlin et al. (8)	Switzerland	RCT	Multicentred RCT	Cryoballon Size: 28 mm Duration of Each Freeze: Time-to-effect plus 2 min strategy (if before 60 s temperature was reached then cryoablation was continued for 2 additional minutes) Bonus Freeze or Not: Not	No	99	102
Tachibana et al. (19)	Japan	Prospective	multi-centred	Cryoballon Size: 28 mm Duration of Each Freeze: 180–240 s Bonus Freeze or Not: Not	No	1,197	1,358

PV, pulmonary vein; RCT, randomized controlled trial.

### 3.2 Meta-analysis of the outcomes

#### 3.2.1 Acute PVI success

**Figure 2:**
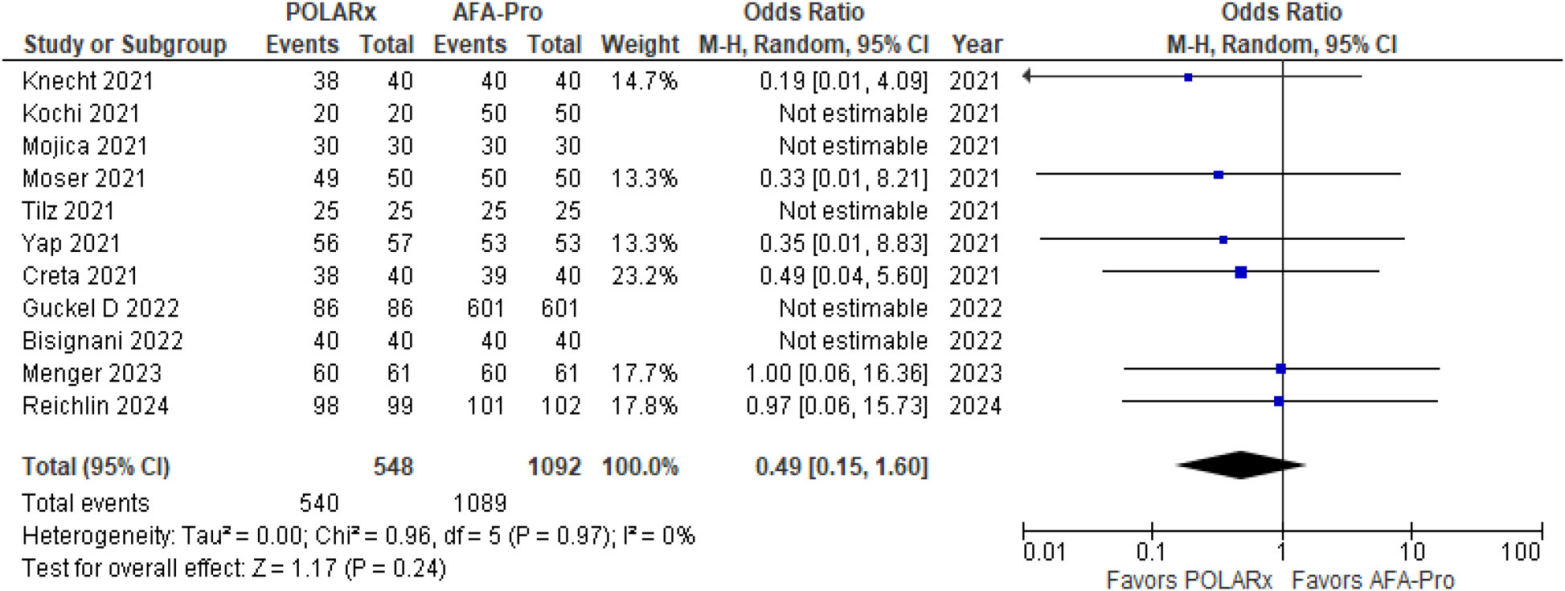
Forest plot illustrating the acute PVI success.

Eleven of the Sixteen reported on acute PVI, with a total of 1,640 participants (548 in the POLARx group and 1,092 in the AFA group). Our analysis showed that both techniques were comparable in terms of acute PVI (OR = 0.49, 95% CI: 0.15–1.60, *P* = 0.24, *I*^2^ = 0%. *Z* = 1.17; Figure 2).

#### 3.2.2 Procedure time

**Figure 3:**
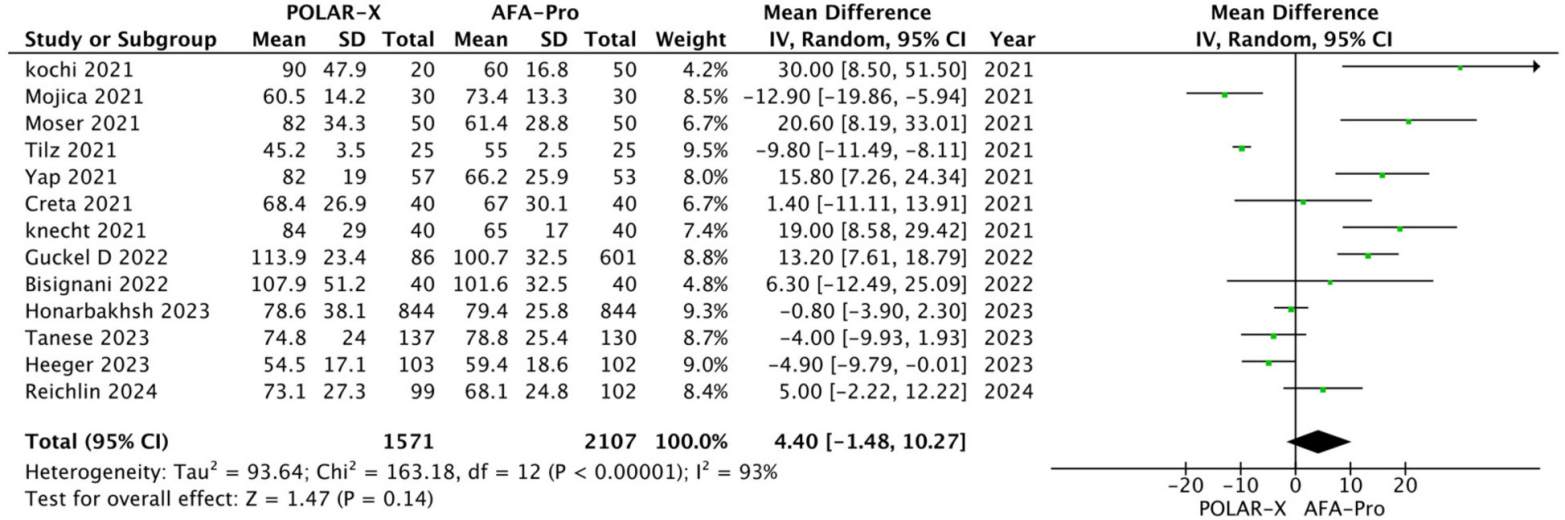
Forest plot illustrating the procedure time.

Thirteen studies reported procedure time, with 3,678 participants (1,571 in the POLARx group and 2,107 in the AFA group). Our analysis indicated that POLARx was comparable to ArcticFront in terms of procedure time (MD = 4.40, 95% CI: −1.48 to 10.27, *P* = 0.14, *I*^2^ = 93%, *Z* = 1.47; Figure 3).

#### 3.2.3 Ablation time

**Figure 4:**
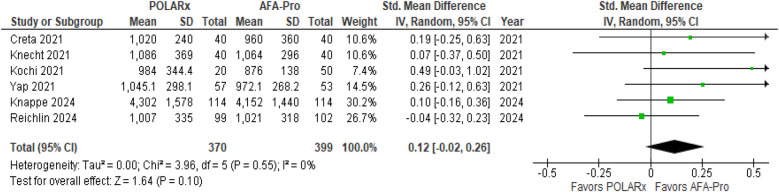
Forest plot illustrating the ablation time.

Six studies evaluated ablation time, and the results showed that there was no notable difference between the two techniques (SMD = 0.12, 95% CI: −0.02 to 0.26, *P* = 0.10, *Z* = 1.64; Figure 4), with negligible heterogeneity (*I*^2^ = 0%).

#### 3.2.4 Fluoroscopy time

**Figure 5:**
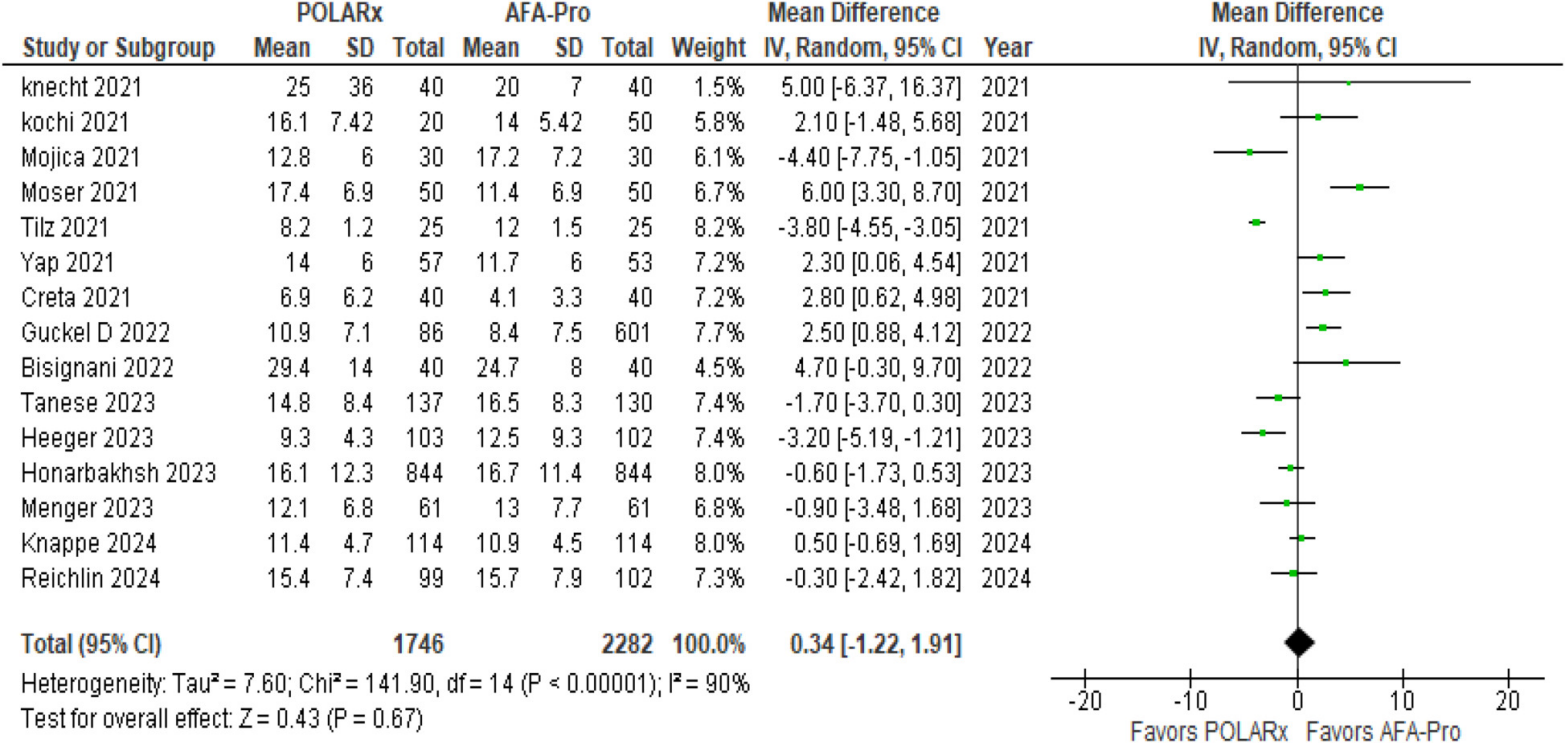
Forest plot illustrating the fluoroscopy time.

Fluoroscopy time was reported in 15 of the 16 studies, involving a total of 4,028 participants (1,746 in the POLARx group and 2,282 in the AFA group). Our analysis revealed that both techniques were comparable in terms of fluoroscopy time (MD = 0.34, 95% CI: −1.22 to 1.91, *P* = 0.67, *I*^2^ = 90%, *Z* = 0.43; Figure 5).

#### 3.2.5 Balloon nadir temperature

**Figure 6:**
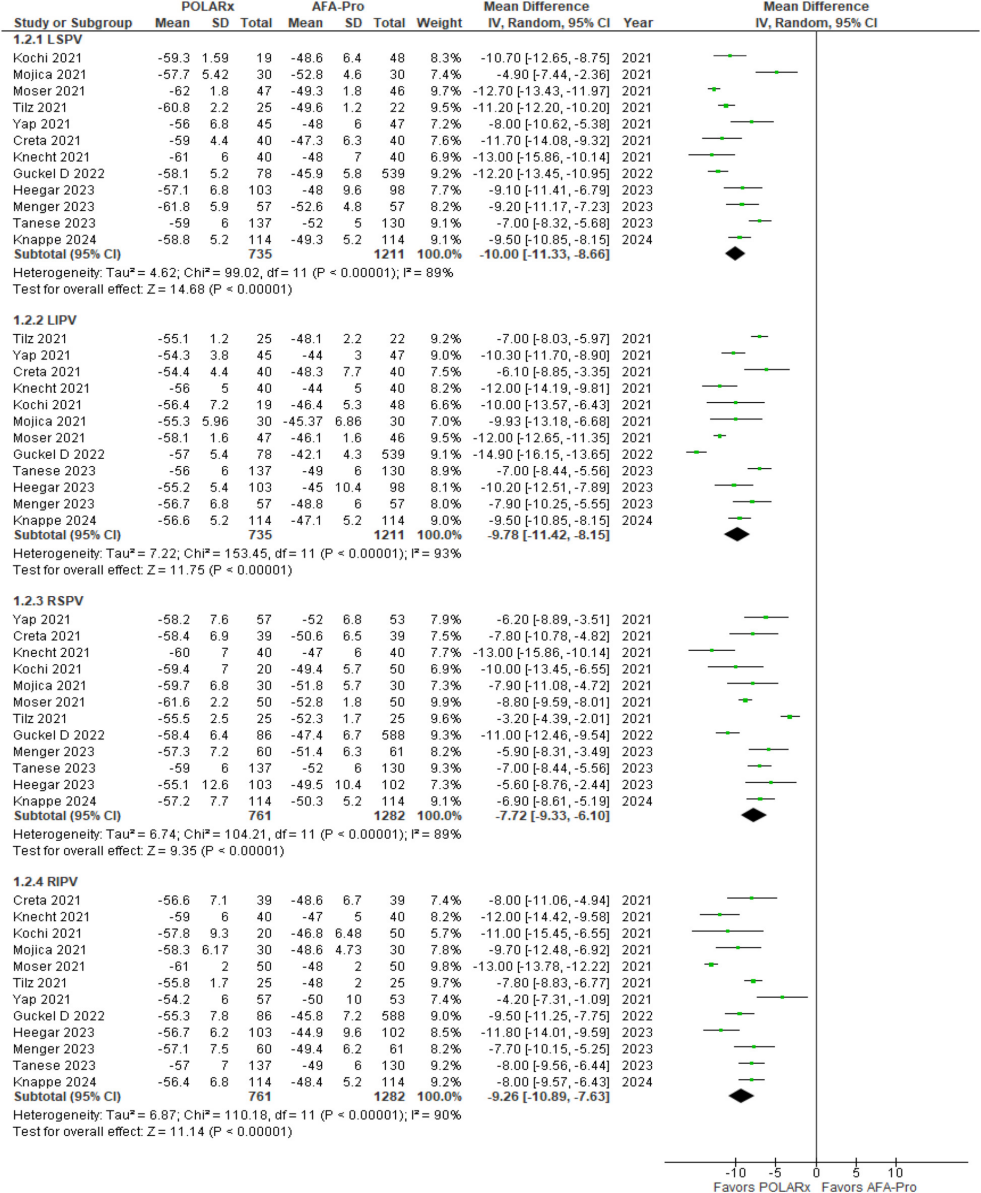
Forest plot illustrating the balloon nadir temperature.

Balloon nadir Temperature was reported in 12 of the 16 studies. Our analysis showed a significant difference favoring POLARx, with marked improvements in balloon nadir temperature (*P* < 0.00001). Specifically, for the left superior pulmonary vein (LSPV), the MD was −10.00 (95% CI: −11.33 to −8.66, *P* < 0.00001, *I*^2^ = 89%, *Z* = 14.68; Figure 6). For the left inferior pulmonary vein (LIPV), the MD was −9.78 (95% CI: −11.42 to −8.15, *P* < 0.00001, *I*^2^ = 93%, *Z* = 11.75; Figure 6). For the right superior pulmonary vein (RSPV), the MD was −7.72 (95% CI: −9.33 to −6.10, *P* < 0.00001, *I*^2^ = 89%; *Z* = 9.35 Figure 6), and for the right inferior pulmonary vein (RIPV), the MD was −9.26 (95% CI: −10.89 to −7.63, *P* < 0. 00001, *I*^2^ = 90%, *Z* = 11.14; Figure 6).

#### 3.2.6 Freezing time

Freezing time data were reported in 5 of the 16 studies. Our analysis showed that both techniques were comparable in terms of freezing time. Specifically, for the LSPV, the freezing time was similar (MD = 7.90, 95% CI: −4.36 to 20.16, *P* = 0.21, *I*^2^ = 22%, *Z* = 1.26). For the LIPV, there was no notable difference (*P* = 0.73), indicating similar efficacy (MD = −3.97, 95% CI: −26.66 to 18.72, *I*^2^ = 59%, *Z* = 0.34). For the RSPV, the analysis showed no clear advantage for POLARx (MD = −5.63, 95% CI: −44.96 to 33.70, *P* = 0.78, *I*^2^ = 91%, *Z* = 0.28), and for the RIPV, there was no marked difference (MD = 1.26, 95% CI: −25.42 to 27.93, *P* = 0.93, *I*^2^ = 73%, *Z* = 0.09; Supplementary Figure 2). Initial Cochran's *Q* test indicated heterogeneity (*I*^2^ = 59%) for LIPV freezing time, but sensitivity analysis reduced it to 21% as shown in Table 3.

**Table 3:** Tabulated results of sensitivity analyses of each heterogeneous outcome.

Outcome	Study excluded	*I*^2^ value before S.A/%	*I*^2^ value after S.A/%	*Z* value (*P*-Value) before S.A/%	*Z* value (*P*-Value) after S.A/%	Mean difference (MD), (95% CI) after SA
Ballon Nadir Temperature LSPV	Tanese et al. (20)	89	84	14.68 (<0.00001)	16.85 (<0.00001)	−10.34 [−11.54, −9.14]
Ballon Nadir Temperature LIPV	Tilz et al. (5)	93	90	11.75 (<0.00001)	12.67 (<0.00001)	−10.08 [−11.64, −8.52]
Ballon Nadir temperature RSPV	Tilz et al. (5)	89	75	9.35 (<0.00001)	13.27 (<0.00001)	−8.21 [−9.42, −7.00]
Ballon Nadir Temperature RIPV	Moser et al. (17)	90	67	11.14 (<0.00001)	15.74 (<0.00001)	−8.82 [−9.92, −7.72]
Freezing Time LSPV	Moser et al. (17)	22	0	1.26 (0.21)	0.01 (0.99)	−0.08 [−15.09, 14.94]
Freezing Time RSPV	Menger et al. (18)	91	71	0.28 (0.78)	1.17 (0.24)	14.48 [−9.73, 38.69]
Freezing Time RIPV	Tanese et al. (20)	73	0	0.09 (0.93)	1.61 (0.11)	15.14 [−3.30, 33.58]
Freezing Time LIPV	Knappe et al. (9)	59	21	0.34 (0.73)	0.4 (0.69)	3.61 [−13.89, 21.10]
TTI recording LSPV	Tilz et al. (5)	87	50	0.11 (0.91)	0.64 (0.52)	1.41 [−2.90, 5.73]
TTI recording LIPV	Knappe et al. (9)	70	38	1.77 (0.08)	4.02 (<0.0001)	6.80 [3.48, 10.12]
TTI recording RSPV	Tilz et al. (5)	78	62	2 (0.05)	1.49 (0.14)	4.00 [−1.25, 9.25]
TTI recording RIPV	Tilz et al. (5)	65	0	2.05 (0.04)	2.28 (0.02)	3.49 [0.49, 6.50]
Procedure Time	Tilz et al. (5)	93	87	1.47 (0.14)	1.93 (0.05)	5.76 [−0.08, 11.59]
Fluoroscopy Time	Tilz et al. (5)	90	80	0.43 (0.67)	0.98 (0.33)	0.65 [−0.65, 1.96]

LSPV, left superior pulmonary vein; LIPV, left inferior pulmonary vein; RSPV, right superior pulmonary vein; RIPV, right inferior pulmonary vein; TTI, time to isolation; S.A, sensitivity analysis; *I*^2^, I-squared (heterogeneity measure); *Z*, *Z*-score; *P*-value, probability value; MD, mean difference; CI, confidence interval.

#### 3.2.7 Time to isolation (TTI)

Nine studies compared TTI, with a total of 1,036 participants (509 in the POLARx group and 527 in the AFA group). The analysis showed that both techniques were comparable in terms of TTI. For the LSPV, the MD was −0.37 (95% CI: −6.67 to 5.93, *P* = 0.91, *I*^2^ = 87%, *Z* = 0.11; Supplementary Figure 3). For the LIPV, TTI was similar between the techniques (MD = 4.21, 95% CI: −0.46 to 8.88, *P* = 0.08, *I*^2^ = 70%, *Z* = 1.77; Supplementary Figure 3). For the RSPV, there was a clear advantage for AFA-PRO (MD = 5.49, 95% CI: 0.11–10.88, *P* = 0.05, *I*^2^ = 78%, *Z* = 2.00; Supplementary Figure 3), with a similar benefit seen for the RIPV (MD = 4.54, 95% CI: 0.21–8.87, *P* = 0.04, *I*^2^ = 65%, *Z* = 2.05; Supplementary Figure 3).

#### 3.2.8 Phrenic nerve palsy

**Figure 7:**
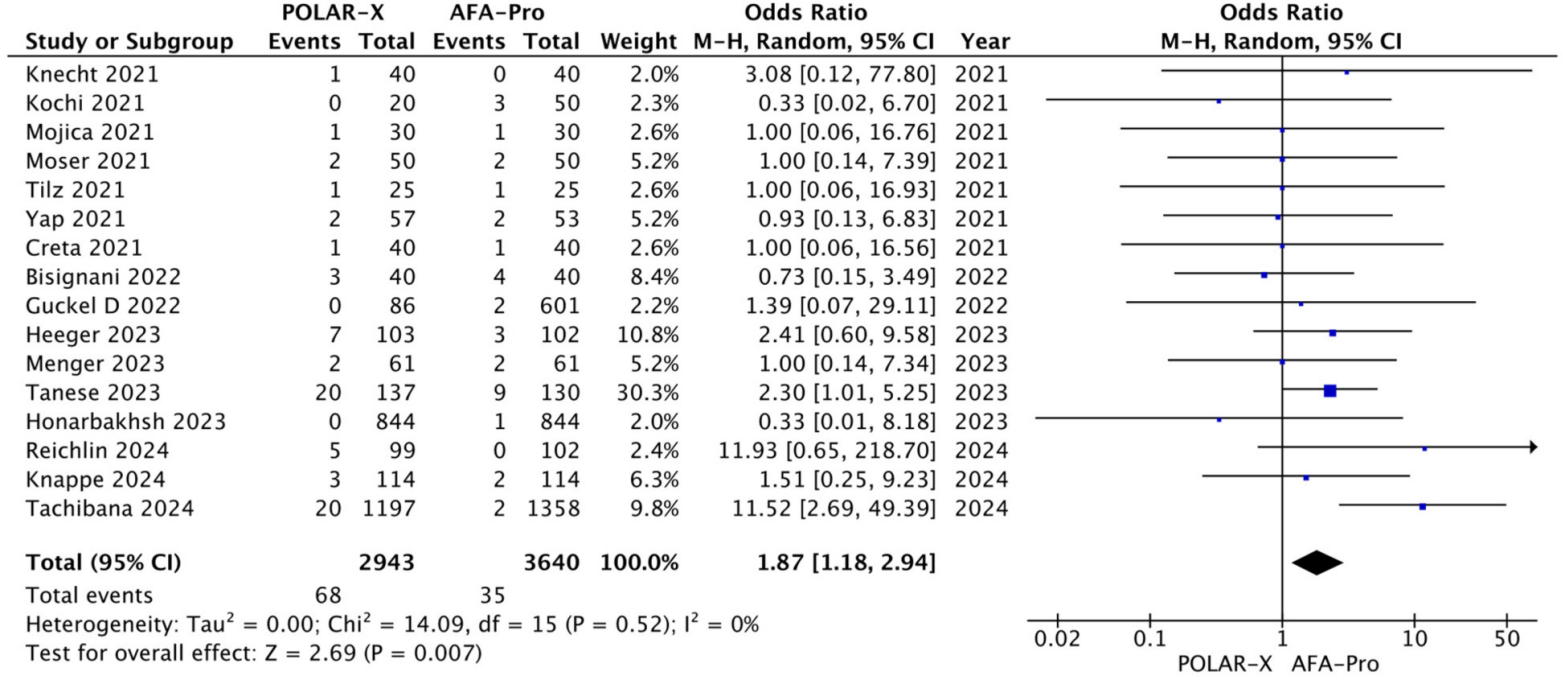
Forest plot illustrating the incidence of phrenic nerve palsy.

Phrenic nerve palsy was reported in all the studies, involving 6,583 participants (2,943 in the POLARx group and 3,640 in the AFA group). Our analysis showed that POLARx was associated with a higher risk of phrenic nerve palsy (OR = 1.87, 95% CI: 1.18–2.94, *P* = 0.007, *I*^2^ = 0%, *Z* = 2.69; Figure 7).

#### 3.2.9 Stroke

Nine studies reported on stroke outcomes, and our analysis found that both techniques were comparable in terms of stroke rates (OR = 3.58, 95% CI: 0.58–22.26; *P* = 0.17, *Z* = 1.49; Supplementary Figure 4). The heterogeneity was minimal (*I*^2^ = 0%).

### 3.3 Risk of bias assessment of included studies

Specifically, three studies (Mojica et al., Guckel et al., Honarbakhsh et al., and Tachibana et al.) achieved the highest score of 8, reflecting well-defined cohorts, sufficient follow-up duration, and robust outcome assessment as evaluated by NOS. A total of 10 studies were rated as high quality (score 7–8), while 4 studies received scores of 5–6, indicating moderate quality. Areas commonly lacking across studies included documentation of adequate follow-up duration and loss to follow-up information. The COMPARE CRYO Randomized trial showed an overall low risk of bias. Randomization and outcome assessment were appropriately blinded, with objective data collection via implantable cardiac monitors. Minor concerns related to lack of clinician blinding were unlikely to impact outcomes (8). Elaborate findings of each study are further presented in Supplementary Table 2, Figures 1A, 1B.

### 3.4 Sensitivity analysis

The sensitivity analysis showed a significant reduction in heterogeneity for certain outcomes after excluding specific studies. For instance, Freezing Time for RIPV exclusion of Tanese et al., 2023 decreased from 73% to 0%, and TTI Recording for RIPV removal of Tilz et al., 2021 dropped from 65% to 0%. Freezing Time for LIPV (exclusion of Knappe et al., 2024) also reduced from 59% to 21%, as illustrated in Supplementary Figures 5 and 6. However, several outcomes showed no significant reduction in heterogeneity after excluding a single study, including balloon nadir temperature for LSPV, balloon nadir temperature for LIPV, procedure time, fluoroscopy time, balloon nadir temperature for RSPV, and TTI recording for LSPV. Importantly, many outcomes also showed an increase in *Z*-values after exclusion, reflecting a stronger overall effect. For instance, the *Z*-value for Balloon Nadir Temperature (LSPV) rose from 14.68 to 16.85 after excluding Tanese et al., and Procedure Time showed improved effect strength (*Z* = 1.93 vs. 1.47) after removing Tilz et al. These findings suggest that some heterogeneity was likely due to differences in freezing protocols, balloon systems, or study-level variability rather than random error alone. The summary of the sensitivity analysis results is detailed in Table 3.

### 3.5 Publication bias

We evaluated potential publication bias across multiple outcomes using both Egger's regression test and Begg's rank correlation test. No significant funnel plot asymmetry was detected for most outcomes. Phrenic Nerve Palsy (Egger's *p* = 0.1477; Begg's *p* = 0.2496), Acute PVI Success (Egger's *p* = 0.5002; Begg's *p* = 0.4951), and all balloon nadir temperature outcomes including LIPV (Egger's *p* = 0.4734), RSPV (*p* = 0.9920), and RIPV (*p* = 0.2241) showed no evidence of asymmetry. However, Procedure Time demonstrated significant funnel plot asymmetry (Egger's *t* = 3.2415, *df* = 10, *p* = 0.0088), with a large negative limit estimate (*b* = −12.0260; 95% CI: −18.3466 to −5.7054), suggesting potential small-study effects. Fluoroscopy Time also showed a significant result (Egger's *p* = 0.0322), with a corresponding limit estimate of *b* = −3.8988 (95% CI: −6.8080 to −0.9896). Although Begg's tests for these outcomes were not statistically significant (Procedure Time *p* = 0.4590; Fluoroscopy *p* = 0.3880), the Egger's results may indicate possible publication bias or small-study effects, particularly for Procedure Time. Balloon Temp in LSPV showed marginal asymmetry (Egger's *p* = 0.0552), though it did not reach conventional significance. A summary of all results is presented in Supplementary Table 3 and Figures S7A–S7E.

## 4 Discussion

This meta-analysis examined 16 studies with 6,583 participants (4–6, 8, 9, 16–26) to assess the efficacy and safety of two cryoballoon ablation systems. Overall, the groups did not differ significantly regarding acute PVI, procedure time, ablation time, fluoroscopy time, or freezing time. Notably, POLARx demonstrated much lower balloon nadir temperature in all pulmonary veins, with the LSPV showing the most significant difference. Safety profiles appeared similar with the exception of higher PNP with PolarX.

Earlier meta-analyses in 2021 and 2022 gave helpful information about the relative efficacy of these systems. The 2021 meta-analysis, which included four trials and 310 patients, found no significant differences in primary procedural outcomes, indicating procedural equivalency (28). The 2022 meta-analysis, spanning eight investigations and 1,146 patients, corroborated these findings, adding that POLARx regularly had lower nadir temperatures, notably in RIPV, and had a prolonged TTI in LIPV, indicating potentially better isolation capabilities (7). However, those investigations were constrained in sample sizes and focused on select pulmonary veins, overlooking comprehensive temperature and TTI comparisons. They also lacked detailed analysis of phrenic nerve palsy and device-specific design factors. This updated meta-analysis addresses these gaps by including a larger dataset, evaluating all pulmonary veins, and offering deeper insight into safety and procedural nuances.

The AFA-Pro cryoballoon is frequently used to obtain PVI because it produces effective and homogenous lesions with low arrhythmogenic potential. POLARx has similar qualities, such as a double-layer balloon design and nitrous oxide cooling. Still, it also has a unique design that maintains inner balloon pressure while freezing, making it more compliant (4). This design may allow for more precise lesion development, but also adds a higher learning curve due to its innovative cryoconsole and steerable sheath. This intricacy may explain why certain studies, such as Reichlein et al.'s, reported higher procedure times with POLARx (8). However, we did not find a significant difference in procedure or ablation times between the two systems, aligning with previous meta-analyses.

A notable finding in this study was that POLARx resulted in much lower balloon nadir temperatures, consistent with previous research (5, 18, 24, 29). It is crucial to note that the inner balloon temperature does not always correspond to the surface temperature, as thermocouple position, energy transfer efficiency, balloon depth within the pulmonary vein, and tissue contact area can all influence temperature readings (6, 27). For POLARx, nadir temperatures as low as −53.5 °C (24) or even −56 °C (30) have been linked to acute and sustained PVI success. These findings suggest that the predictors of lesion durability may need to be adjusted for POLARx's lower nadir temperatures and faster cooling profiles.

TTI serves as an essential marker of procedural efficiency in PVI. Previous studies suggested that POLARx had prolonged TTI for the LIPV due to catheter positioning and balloon stability. Our analysis comprehensively evaluated TTI across all pulmonary veins, finding no significant differences in TTI for the LSPV or LIPV. However, there was a notable increase in TTI for the RSPV and RIPV with POLARx. Variations in TTI may be attributed to anatomical differences, as left-sided pulmonary veins tend to have more uniform structures, facilitating energy delivery. In contrast, right-sided veins have variable orientations that could challenge energy delivery and prolong TTI (31). The POLARx system's design, balloon compliance, and cooling mechanisms might improve lesion formation in specific veins. However, operator-dependent factors like optimal contact force and positioning and the proximity of the phrenic nerve to the right-sided veins could further complicate the procedure and affect TTI outcomes. Despite these factors, freezing times were similar between both systems, suggesting comparable procedural cooling efficacy.

Our meta-analysis revealed a increased risk of PNP linked with the POLARx system. This could be explained by POLARx's lower temperatures, which could pose a greater risk to surrounding structures. The POLARx system's DMS™ sensor detects partial PNP with more sensitivity than the AFA-Pro's tactile feedback, perhaps leading to a higher detection rate of transitory PNP (20). The POLARx balloon's compliant design may also allow for deeper advancement into the pulmonary veins, potentially bringing it closer to the phrenic nerve especially in the right-sided veins, where the nerve's anatomical course is more variable (32). Although the majority of phrenic nerve palsy cases are temporary and resolve within weeks to months, persistent dysfunction though relatively uncommon, can result in unilateral diaphragmatic paralysis. This condition may lead to symptoms such as shortness of breath, orthopnea, and decreased exercise tolerance, particularly in individuals with pre-existing pulmonary or cardiac conditions (33). Techniques such as continuous diaphragmatic pacing and compound motor action potential (CMAP) monitoring have been recommended to allow early detection of phrenic nerve impairment and prevent permanent injury (34). To mitigate this risk, clinicians should utilize real-time imaging to ensure optimal balloon positioning, apply electrophysiological mapping to assess phrenic nerve proximity, and consider next-generation devices such as the POLARx Fit, which offers more precise balloon sizing. Moreover, refining freezing techniques and enhancing operator familiarity with the POLARx system may further reduce the likelihood of phrenic nerve injury.

In addition to safety outcomes, long-term efficacy remains a critical aspect of cryoballoon ablation performance. In a study by Tanese et al., atrial fibrillation (AF) recurrence rates during the 3-month blanking period were similar between the AFA-Pro and POLARx systems. Over a mean follow-up of approximately 15–16 months, a slightly higher number of patients in the POLARx group experienced AF recurrence beyond the blanking period; however, by one year post-procedure, the proportion of patients without recurrent AF episodes was comparable across both groups. Interestingly, a greater number of patients treated with POLARx underwent repeat ablation procedures during follow-up, though this did not appear to reflect a higher overall recurrence burden. All repeat procedures were performed using radiofrequency energy rather than cryoballoon technology (20). These findings suggest that both systems provide similar long-term rhythm control, while the increased rate of reintervention in the POLARx group may reflect factors such as the learning curve, patient anatomy, or operator preferences, rather than inherent differences in device efficacy.

### 4.1 Clinical implications

Our meta-analysis findings provide clinicians with essential recommendations. Because POLARx has lower balloon nadir temperatures, it may be particularly helpful for people who have repeated AF following previous ablation. This could lead to more permanent lesions and a decreased risk of arrhythmia recurrence. The higher risk of phrenic nerve palsy associated with POLARx may call for more cautious treatment in patients with anatomical issues, such as fluctuating right-sided pulmonary vein placements, particularly when treating veins close to the phrenic nerve. POLARx system's lower nadir temperatures and greater balloon compliance may offer advantages in patients with complex or variable pulmonary vein anatomy, potentially improving lesion formation and procedural success. Conversely, the AFA-Pro system may be more suitable for patients with elevated risk of PNP, such as those with prior thoracic surgeries, baseline diaphragmatic weakness, or significant pulmonary disease, given its lower observed PNP incidence. Using AFA-Pro or changing the ablation technique can help lower the chance of issues in certain situations. POLARx may reduce the need for follow-up treatments by improving lesion lifespan in younger individuals or those with a higher arrhythmia load.

Furthermore, for patients with left atrial enlargement or fibrosis, where long-lasting PVI is more problematic, POLARx's superior tissue cooling may aid in forming deeper and more consistent lesions, enhancing long-term outcomes. POLARx may improve procedural results in facilities with experienced operators, particularly for high-risk arrhythmia patients, as long as phrenic nerve injury is adequately controlled. Finally, the cryoballoon system should be tailored to each patient's anatomical characteristics, arrhythmia burden, and clinical experience to provide the highest level of safety and efficacy.

### 4.2 Limitations

Even though our meta-analysis provides insightful information, there are a few significant limitations to be aware of. The pooled results may have been limited in generalizability due to heterogeneity in study design, sample size, operator experience, and procedural protocols. The variability in freezing techniques (e.g., freeze duration, use of bonus applications), balloon generation used, and procedural workflows (e.g., time-to-isolation strategy, balloon positioning) may have contributed to statistical heterogeneity observed in certain outcomes. Because no single study had a substantial impact on effect sizes, a leave-one-out sensitivity analysis validated the reliability of the results. Moreover, in our assessment of Publication bias assessment, Egger's test revealed possible publication bias for procedure time and fluoroscopy time. Although Begg's test did not confirm this, the small number of included studies limits the power of these assessments. Therefore, findings related to these parameters should be interpreted with caution. Detailed comparisons were further hampered by inconsistent reporting of essential characteristics, such as freezing time and nadir temperature. Future relevance may also be impacted by the POLARx system's challenging learning curve and quick technical developments, such as the POLARx Fit.

## 5 Conclusion

This meta-analysis concludes that the POLARx and AFA-Pro cryoballoon systems show comparable efficacy and safety profiles in pulmonary vein isolation procedures. Nonetheless, POLARx is linked to lower balloon nadir temperatures and a slightly increased incidence of phrenic nerve palsy, especially in veins on the right side. Although both systems work well, doctors should choose the right one based on patient characteristics, operator experience, and anatomical considerations. Future research should concentrate on large-scale, multicenter randomized studies to validate these findings, evaluate long-term outcomes, and investigate newer device iterations for enhanced procedural efficiency and safety.

## Data Availability

The original contributions presented in the study are included in the article/Supplementary Material, further inquiries can be directed to the corresponding author.
