# Supplementary material for: Comparative efficacy and safety of POLARx versus Arctic front advance pro cryoballoon systems for pulmonary vein isolation in atrial fibrillation: an updated systematic review and meta-analysis
Source: Front Cardiovasc Med. 2025 Aug 18;12:1625399. doi: 10.3389/fcvm.2025.1625399 (PMC12400518; doi:10.3389/fcvm.2025.1625399)
Supplement: Supplementary file 1 [file Datasheet1.docx]

| **Database** | **String** | **Results** |
| --- | --- | --- |
| **PubMed** | ("cryoablated"[All Fields] OR "cryosurgery"[MeSH Terms] OR "cryosurgery"[All Fields] OR "cryoablation"[All Fields] OR "cryoablations"[All Fields] OR ("cryoballoon"[All Fields] OR "cryoballoons"[All Fields]) OR ("fourth-generation"[All Fields] OR "4th-generation"[All Fields] OR ("4th"[All Fields] AND ("curr biol"[Journal] OR "cb"[All Fields])) OR "CB4"[All Fields] OR "CBG4"[All Fields] OR (("arctic regions"[MeSH Terms] OR ("arctic"[All Fields] AND "regions"[All Fields]) OR "arctic regions"[All Fields] OR "arctic"[All Fields] OR "arctic s"[All Fields]) AND ("front"[All Fields] OR "front s"[All Fields] OR "fronts"[All Fields])) OR ("alanylproline"[Supplementary Concept] OR "alanylproline"[All Fields] OR "ala pro"[All Fields]) OR "POLARx"[All Fields])) AND ((("pulmonary veins"[MeSH Terms] OR ("pulmonary"[All Fields] AND "veins"[All Fields]) OR "pulmonary veins"[All Fields] OR ("pulmonary"[All Fields] AND "vein"[All Fields]) OR "pulmonary vein"[All Fields]) AND ("isolate"[All Fields] OR "isolate s"[All Fields] OR "isolated"[All Fields] OR "isolates"[All Fields] OR "isolating"[All Fields] OR "isolation and purification"[MeSH Subheading] OR ("isolation"[All Fields] AND "purification"[All Fields]) OR "isolation and purification"[All Fields] OR "isolation"[All Fields] OR "isolations"[All Fields])) OR "PVAI"[All Fields] OR "PVI"[All Fields]) AND ("atrial fibrillation"[MeSH Terms] OR ("atrial"[All Fields] AND "fibrillation"[All Fields]) OR "atrial fibrillation"[All Fields] OR ("paroxysmal"[All Fields] AND "atrial"[All Fields] AND "fibrillation"[All Fields]) OR "paroxysmal atrial fibrillation"[All Fields]) | 1529 |
| **Google Scholar** | (cryoablation OR cryoballoon) AND (fourth-generation OR 4th-generation OR 4th- CB OR CB4 OR CBG4 OR arctic front OR ala pro OR POLARx) AND (pulmonary vein isolation OR PVAI OR PVI) AND (paroxysmal Atrial fibrillation) | 166 |
| **Science direct** | (cryoablation OR cryoballoon) AND (pulmonary vein isolation OR PVAI OR PVI) AND (paroxysmal Atrial fibrillation) | 660 |
| **Clinical trials.gov** | Paroxysmal Atrial Fibrillation \| Other terms: Pulmonary Vein Isolation \| Cryoballoon Ablation | 37 |

**Supplementary Table 1:** Detailed search strategy.

**Supplementary Table 2:** The Newcastle-Ottawa Scale for assessing the quality of nonrandomized studies in the meta-analysis.

|  | **Selection** | | | | **Comparability of the Cohort** | **Outcome** | | | **Total Score** |
| --- | --- | --- | --- | --- | --- | --- | --- | --- | --- |
| **Study, Year** | **Representativeness of the Exposed Cohort** | **Selection of the Non-Exposed Cohort** | **Ascertainment of Exposure** | **Outcome Not Present at Baseline** |  | **Assessment of Outcome** | **Enough Follow-Up Duration** | **Adequate Follow-Up** |  |
| **Kochi, 2021** | * | * | * | * | - | * | - | * | 6 |
| **Creta, 2021** | * | * | * | * | * | - | - | * | 6 |
| **Moser, 2021** | * | * | * | * | - | * | - | - | 5 |
| **Mojica, 2021** | * | * | * | * | ** | * | * | - | 8 |
| **Yap, 2021** | * | * | * | * | * | * | - | * | 7 |
| **Tilz, 2021** | * | * | * | * | * | * | * | - | 7 |
| **Sven Knecht, 2021** | * | * | * | * | - | * | - | * | 6 |
| **Bisignani, 2022** | * | * | * | * | * | * | - | * | 7 |
| **Denise Guckel, 2022** | * | * | * | * | * | * | * | * | 8 |
| **Tachibana 2024** | * | * | * | * | * | * | * | * | 8 |
| **CH Heeger, 2023** | * | * | * | * | * | * | - | * | 7 |
| **Menger, 2023** | * | * | * | * | * | * | - | * | 7 |
| **Honarbakhsh, 2023** | * | * | * | * | * | * | * | * | 8 |
| **Tanese, 2023** | * | * | * | * | ** | - | * | * | 7 |
| **Knappe, 2024** | * | * | * | * | ** | * | - | - | 7 |

Each asterisk represents one star in the Newcastle-Ottawa Scaling System. The maximum stars are 2 for comparability and 1 are for all other categories. Each star counts towards the total score. Score of 5 to 6 is considered as moderate quality and 7 to 9 as high quality.

- Not available or unable to extract.

**Supplementary Table 3:**Assessment of Publication Bias Using Egger’s and Begg’s Tests for Selected Outcomes

| **Outcome** | **Egger’s Test (t, df, p)** | **Limit Estimate (b, 95% CI)** | **Begg’s Test (Kendall’s tau, p)** |
| --- | --- | --- | --- |
| Phrenic Nerve Palsy | t = -1.5392, df = 13, p = 0.1477 | b = 1.3731 (CI: 0.2063, 2.5400) | τ = -0.2727, p = 0.2496 |
| Acute PVI Success | t = -0.7645, df = 3, p = 0.5002 | b = 1.2296 (CI: -6.1738, 8.6329) | τ = -0.1429, p =0.4951 |
| Procedure Time | t = 3.2415, df = 10, p = 0.0088 | b = -12.0260 (CI: -18.3466, -5.7054) | \| τ = 0.1818 \| \| --- \|  \|  \| \| --- \|   , p =0.4590 |
| Fluoroscopy | t = 2.4221, df = 12, p = 0.0322 | b = -3.8988 (CI: -6.8080, -0.9896) | τ = 0.1868, p = 0.3880 |
| Balloon Temp - LSPV | t = 2.2010, df = 9, p = 0.0552 | b = -13.5607 (CI: -16.6502, -10.4712) | τ = 0.2727, p = 0.2830 |
| Balloon Temp - LIPV | t = 0.7483, df = 9, p = 0.4734 | b = -11.7165 (CI: -15.6225, -7.8104) | τ = -0.0182, p = 1.0000 |
| Balloon Temp - RSPV | t = -0.0104, df = 9, p = 0.9920 | b = -7.6015 (CI: -11.7149, -3.4880) | τ = -0.0909, p = 0.7612 |
| Balloon Temp - RIPV | t = 1.3057, df = 9, p = 0.2241 | b = -12.0862 (CI: -15.8349, -8.3375) | τ = -0.1273, p = 0.6481 |

**Supplementary Figure 1A:** Risk of Bias Graph.


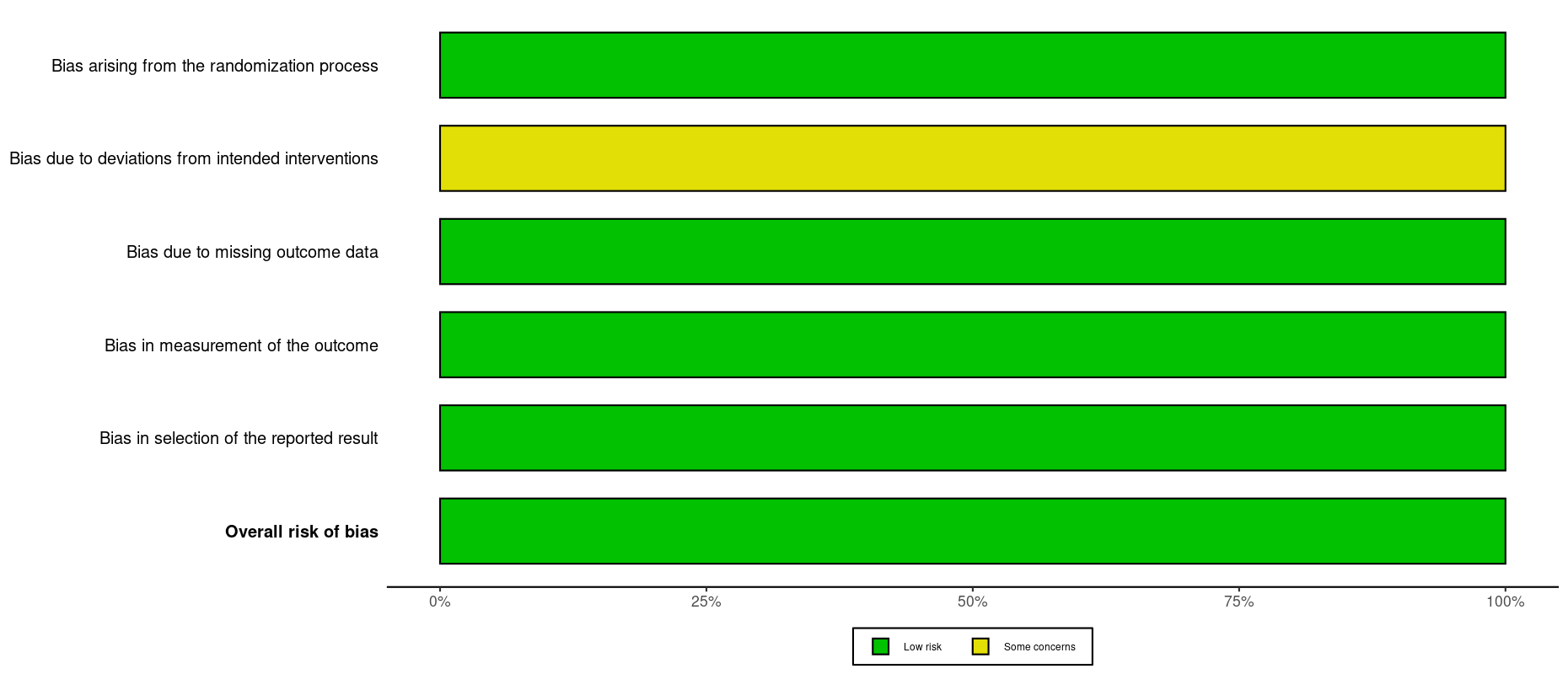


**Supplementary Figure 1B:** Risk of Bias summary.


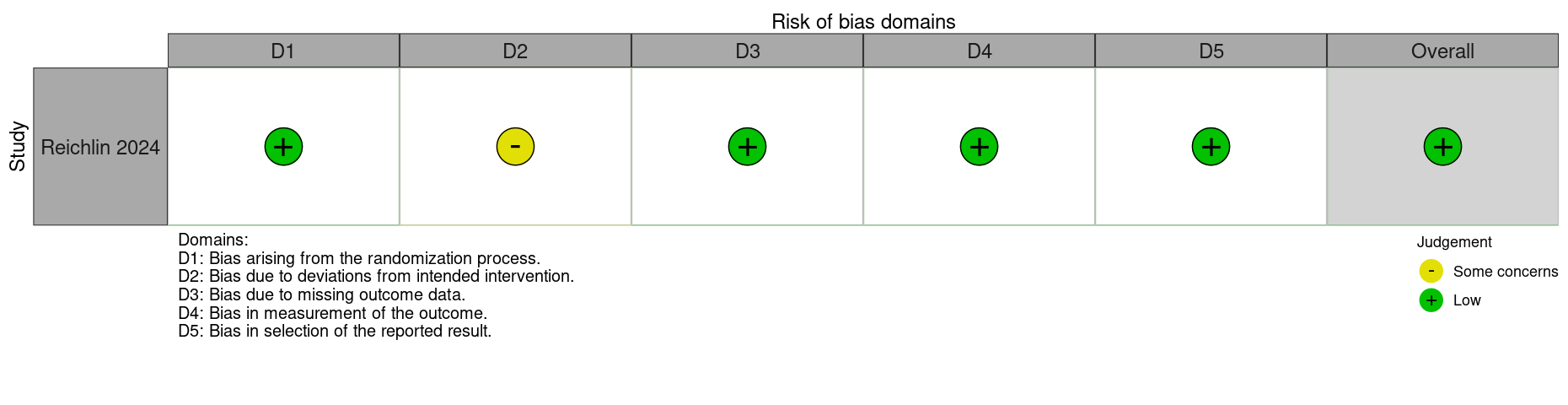


**Supplementary Figure 2:** Forest plot illustrating the freezing time.


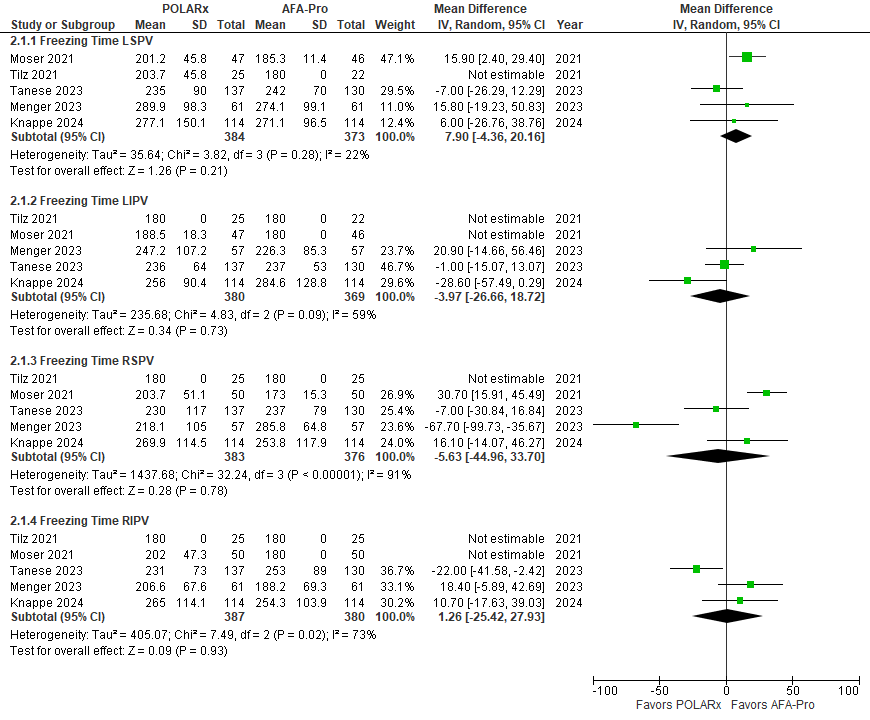


**Supplementary Figure 3:** Forest plot illustrating the TTI Recording.


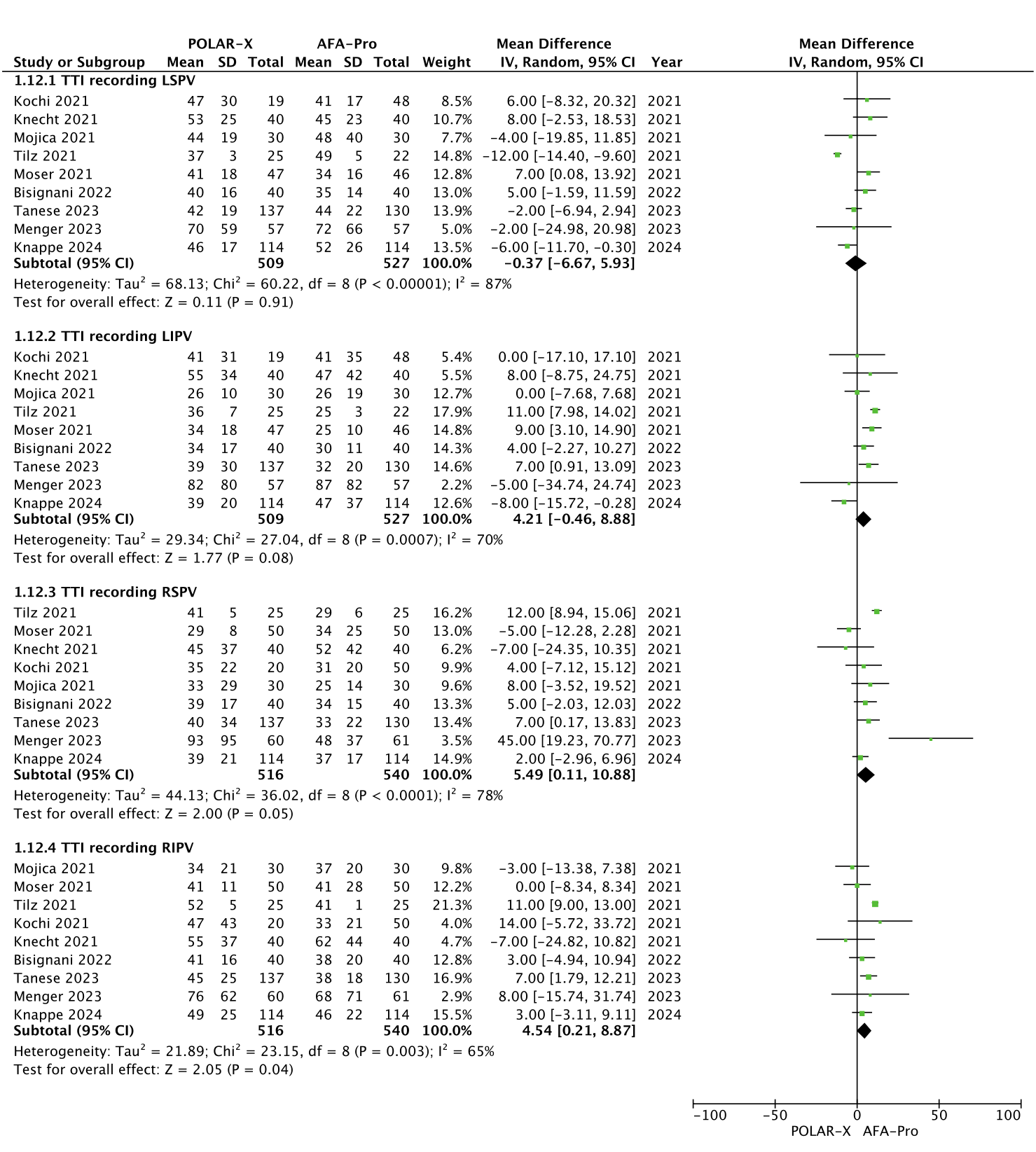


**Supplementary Figure 4:** Forest plot illustrating the incidence of stroke.


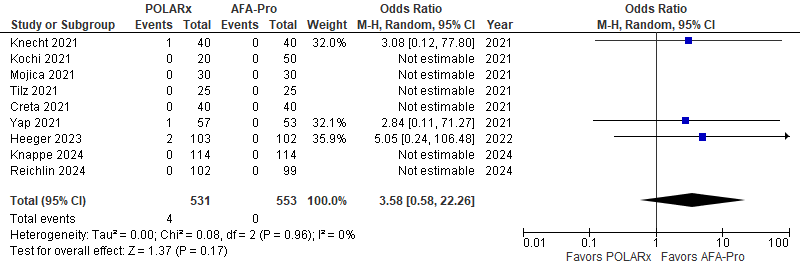


**Supplementary Figure 5:** Post sensitivity analysis forest plot for TTI Recording.


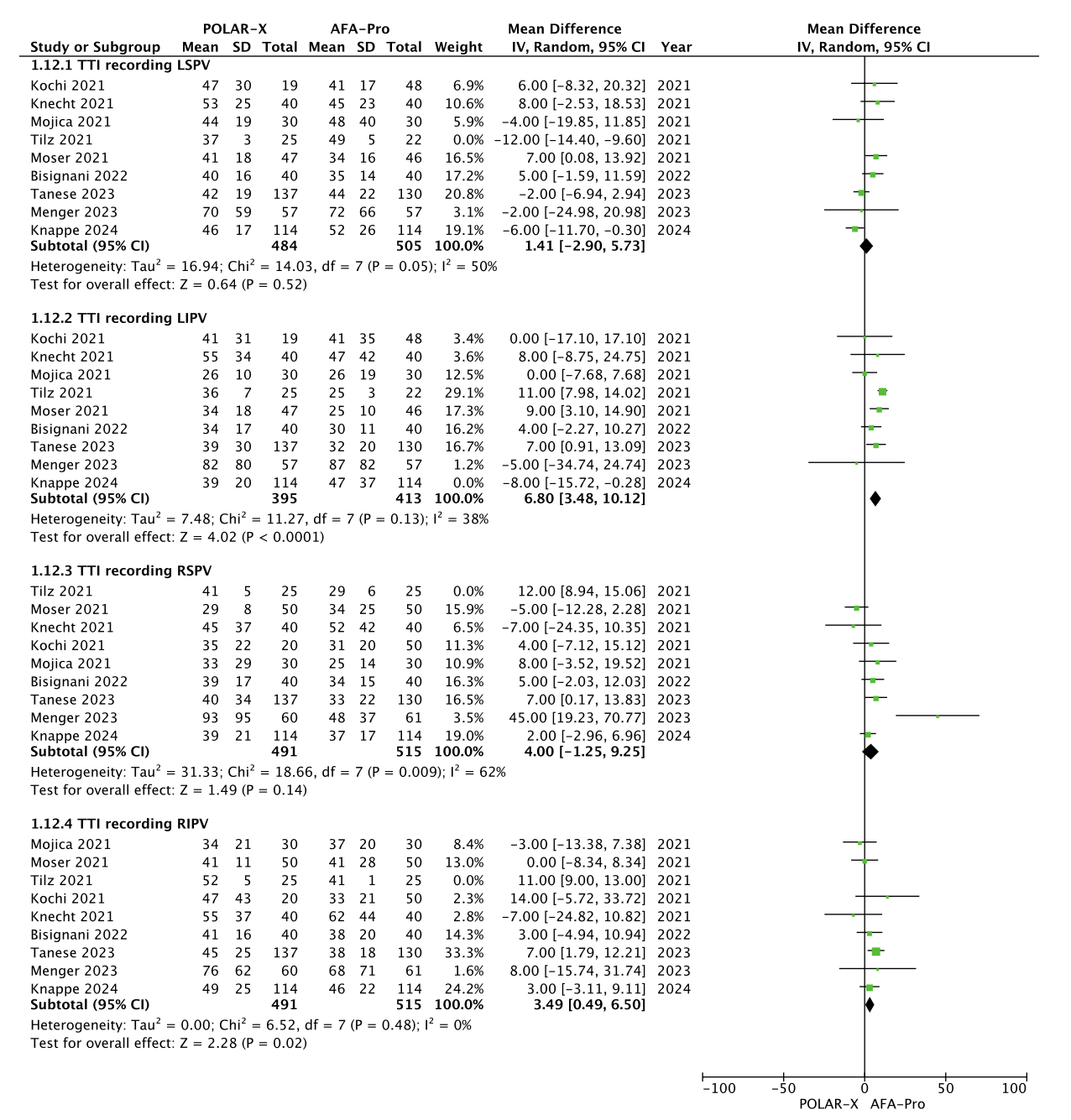


**Supplementary Figure 6:** Post sensitivity analysis forest plot for Freezing Time.

**
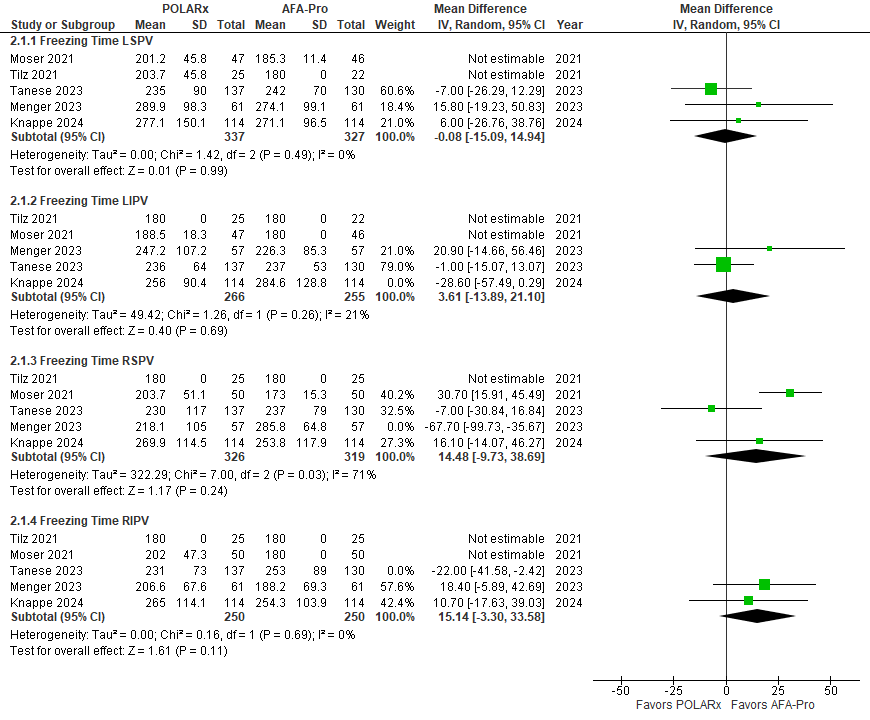
**

**Publication Bias Assessments:**

**Supplementary Figure 7a: Publication Bias Assessment for Phrenic Nerve Palsy**

**
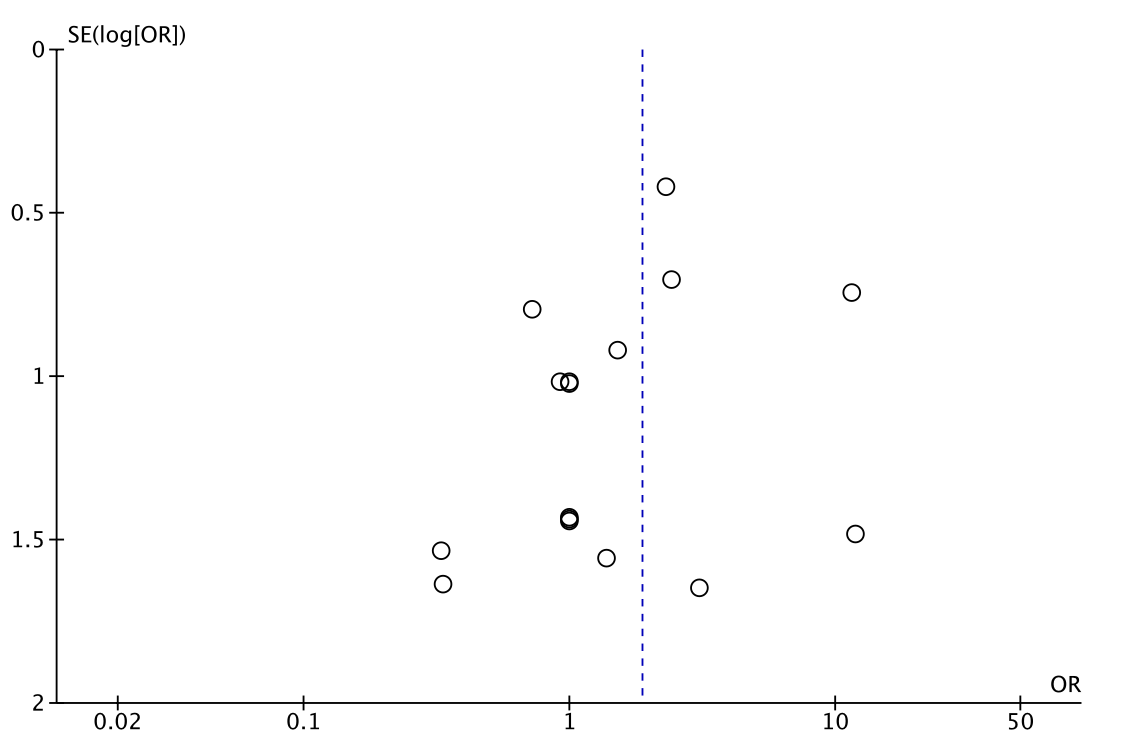
**

**Supplementary Figure 7b: Publication Bias Assessment for Acute PVI Success**

**
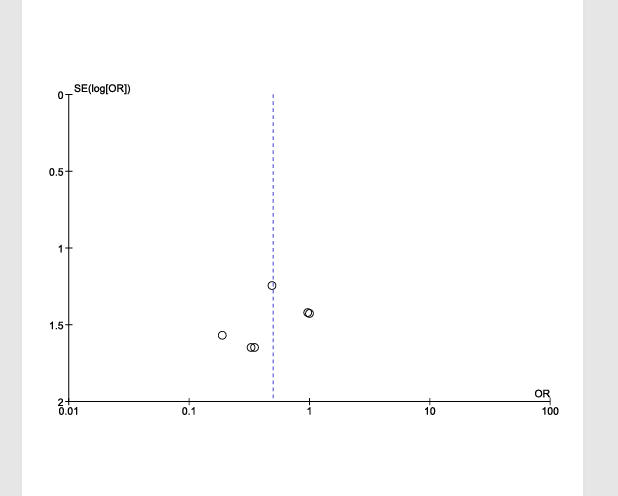
**

**Supplementary Figure 7c: Publication Bias Assessment for Procedure Time**

**
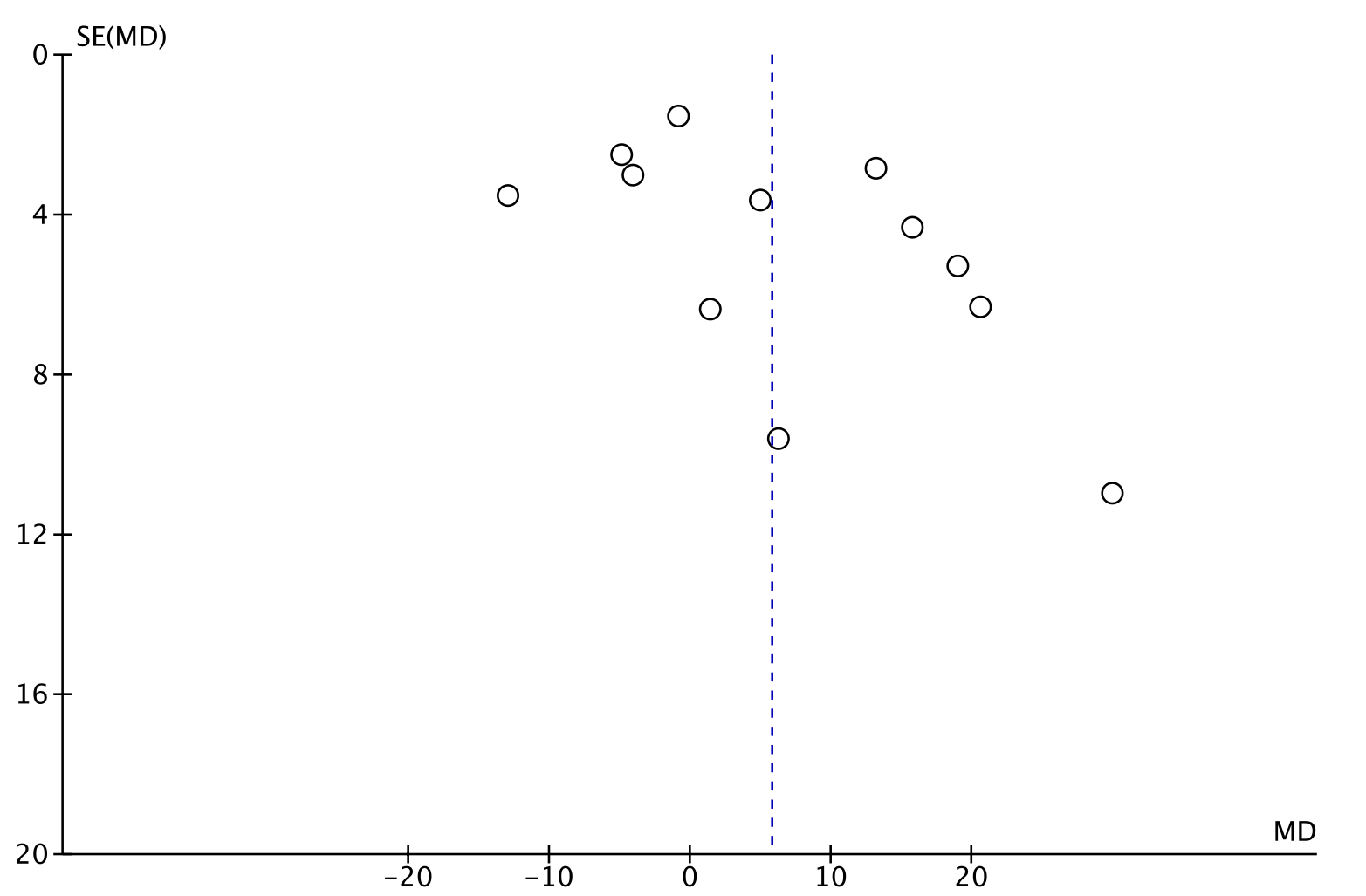
**

**Supplementary Figure 7d: Publication Bias Assessment for Fluoroscopy Time**

**
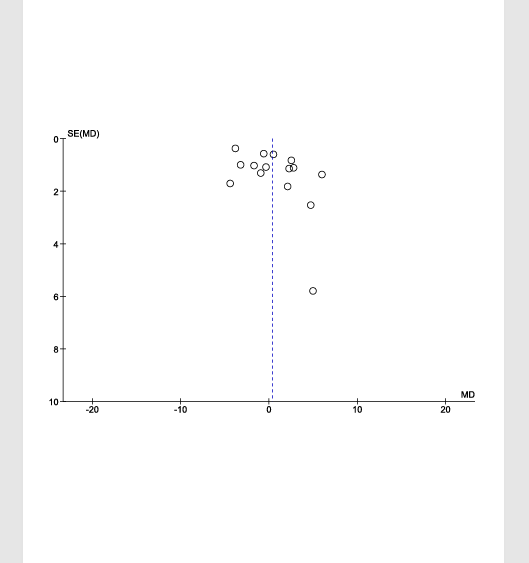
**

**Supplementary Figure 7e: Publication Bias Assessment for Balloon Nadir Temperature**

**
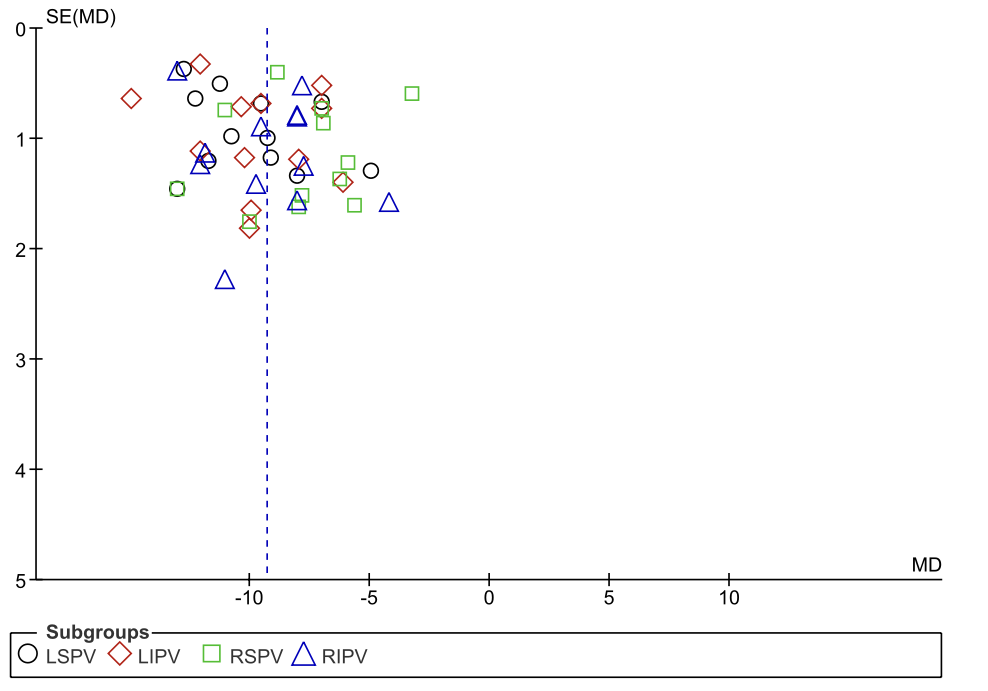
**
